# The Effect of Autologous Platelet-Rich Plasma on Bronchial Stump Tissue Granulation after Pneumonectomy: Experimental Study

**DOI:** 10.1155/2013/864350

**Published:** 2013-12-16

**Authors:** Eleftherios Spartalis, Periklis Tomos, Petros Konofaos, Grigorios Karagkiouzis, Georgia Levidou, Nikolaos Kavantzas, Alkistis Pantopoulou, Othon Michail, Despina Perrea, Gregory Kouraklis

**Affiliations:** ^1^2nd Department of Propedeutic Surgery, Athens General Hospital Laiko, Medical School, University of Athens, Agiou Thoma 17, 11527 Athens, Greece; ^2^Department of Pathology, Medical School, University of Athens, Mikras Asias 75, 11527 Athens, Greece; ^3^Laboratory of Experimental Surgery and Surgical Research “N.S. Christeas,” Medical School, University of Athens, Agiou Thoma 15B, 11527 Athens, Greece

## Abstract

*Objectives*. Recent advances in perioperative management, antibiotics, and surgical materials, including mechanical staplers, have decreased the operative risk of pulmonary resection. However, bronchopleural fistula can still occur in some instances, the occurrence often being lethal. This study investigated whether platelet-rich plasma (PRP) promotes granulation of the bronchial stump after pneumonectomy. *Methods*. Ten pigs were randomized into two groups: (A) control or non-PRP group (pneumonectomy) and (B) PRP group (pneumonectomy and PRP application). PRP was obtained by spinning down the animal's own blood and collecting the buffy coat containing platelets and white blood cells. *Results*. Increased platelet concentration triggered the healing process. The percentage of granulation tissue formed at the stumps was significantly higher in the PRP group of animals. This observation was confirmed when statistical analysis using Mann-Whitney *U* test was performed (*P* = 0.0268). *Conclusions*. PRP is easily produced with minimal basic equipment and is useful in accelerating granulation of the bronchial stump, although the timing and optimum number of applications in humans require further study. Autologous PRP is a safe, feasible, and reliable new healing promoter with potential therapeutic effects.

## 1. Introduction

Since the first pneumonectomy performed by Graham and Singer in 1933 [1], the mortality of this procedure has decreased progressively [2]. The improvements in the surgical technique have contributed significantly to this decrease. Although parallel declines in the incidence of bronchopleural fistula (BPF) have been reported, this complication remains a major concern for the thoracic surgeon with associated high morbidity and mortality rates [3]. Most bronchial stump fistulas appear early after pneumonectomy [3].

One of the promising but clinically challenging areas of therapeutic advances involves the topical application of growth factors to enhance normal healing. A new strategy to better support and promote the wound-healing cascade is to prepare an autologous platelet concentrate suspended in plasma (platelet-rich plasma (PRP)) for application to wound sites in a wide variety of surgeries. The therapeutic effects of PRP are believed to result from the elevated levels of growth factors, for example, platelet-derived growth factor (PDGF) [4].

Using a porcine model, this study evaluated the feasibility of generating PRP with gravitational platelet separation and investigated the granulation process after application on the bronchial stump.

## 2. Material and Methods

### 2.1. The Porcine Model

For our animal study we used a porcine model (sus scrofus domesticus) since the porcine lung is large enough to more accurately simulate the human lung. Porcine models have become an important resource in biomedical and experimental research. The porcine lung has become an excellent model for the normal human lung, for abnormalities in diseases and for therapeutics [5–10].

The pig lung has the dorsal, ventral, medial, and lateral bronchiole systems on either side. In addition, a tracheal bronchus arises from the right side of the trachea (“pig bronchus”) [11]. According to the bronchial ramification, the right lung consists of the cranial, middle, caudal, and accessory lobes, while the left lung consists of the bilobed middle and caudal lobes. The presence of a right tracheal bronchus suggested that left pneumonectomy would be more accurate as part of a human simulation experimental protocol.

Ten male pigs with an average weight of 26 kg were divided and randomized into two groups: the control group or non-PRP group or group A (pneumonectomy) and the PRP group or group B (pneumonectomy and PRP application).

Approval from the Animal Research Ethics Board of the Prefecture of Athens was obtained before commencement of this experimental protocol. All animals received humane care in compliance with the European Convention on Animal Care and in accordance with the National Research Council's criteria (NIH publication number. 86-23, revised 1985). Study was approved by the Institutional Ethics Committee of the National and Kapodistrian University of Athens Medical School. The animals were sacrificed following euthanasia guidelines adapted from the American Veterinary Medical Association Panel on Euthanasia.

### 2.2. Platelet-Rich Plasma Preparation

PRP was easy to produce with minimal effort and prepared as needed at the point of study [12]. It was obtained by spinning down the animal's own blood (23cc) and collecting the buffy coat containing platelets and white blood cells. In a two-step process, whole blood from the animal first centrifuged to separate the plasma from packed red blood cells and then further centrifuged to separate PRP from platelet-poor plasma. This concentrate was then activated with the addition of CaCl, resulting in a gelatinous platelet gel.

Twenty-three milliliters of venous blood were withdrawn from the ear vein before administration of anesthesia. Blood was collected in 5 mL tubes containing 3.8% trisodium citrate and then centrifuged at 1800 rpm for 8 min. The 1 mL fractions located immediately above the erythrocytes were collected from each tube and transferred to sterile tubes. Fifty microliters of CaCl at 10% (w/v) were added per 1 mL fraction of platelet-enriched plasma, and the tubes were put to heat until cylindrical clots were obtained in a few minutes. Clinically valuable PRP contains at least one million platelets per microliter [13]. Lesser concentrations cannot be relied on to enhance wound healing, and greater concentrations have not been shown to increase wound healing [14, 15].

### 2.3. Anesthesia

A single dose of prophylactic cefamandole was given preoperatively. Induction to anesthesia was managed by administration of pentothal 8 mg/kg, pancuronium 0.15 mg/kg, and fentanyl 17.5 *μ*g/kg. Under general anesthesia the animals were placed in the supine position, intubated with a double-lumen endotracheal tube, and mechanically ventilated. Two large-bore vein catheters were placed for intravenous fluid and drug administration. Maintenance to anesthesia was managed by administering of pentothal at 3.5 mg/kg/h, pancuronium at 0.07 mg/kg every 20 minutes, and fentanyl at 15 *μ*g/kg/hr iv. The animals were placed on the right lateral decubitus position. Their vital signs were closely monitored during the operative procedure. Anaesthesia was ended and animals were extubated when sufficient spontaneous breathing was achieved.

### 2.4. Left Pneumonectomy

**Figure 1:**
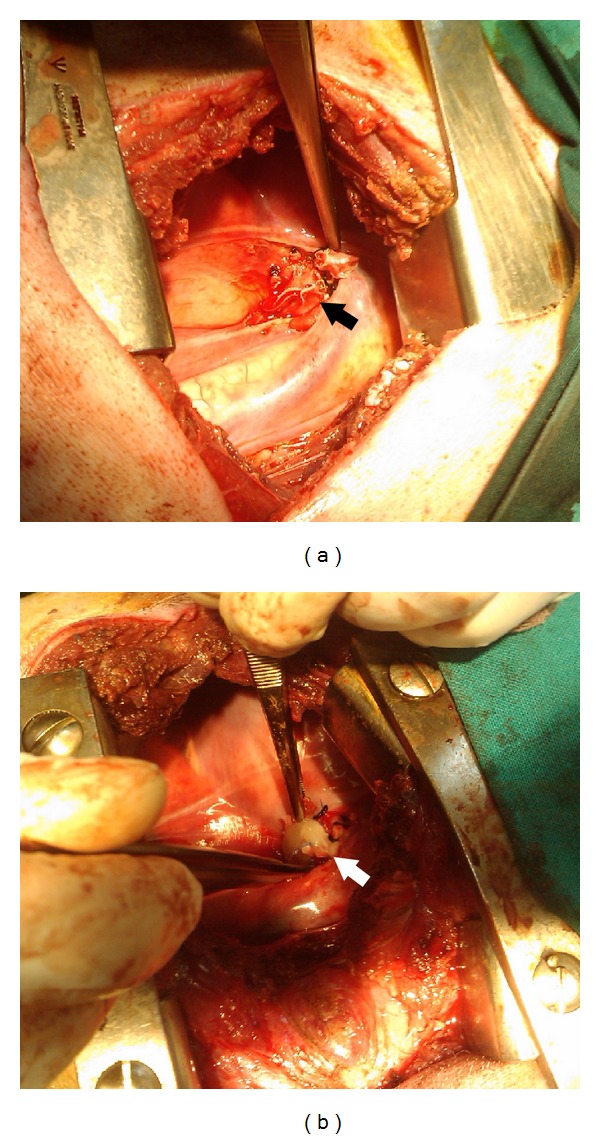
(a) The left bronchus is divided with an endostapler. The black arrow shows the bronchial suture line. (b) The line is covered with a cylindrical clot of PRP applied vertically to the bronchial stump (white arrow).

A standard posterolateral thoracotomy via muscle-sparing incision was performed and the chest entered through the fifth intercostal space. A vascular clamp was placed on the proximal pulmonary artery and the artery was divided and closed with a running silk suture. The superior pulmonary vein was also clamped and sewn as described above. The inferior pulmonary vein was approached from the anterior aspect of the hilum, clamped, and divided. The bronchus was retracted laterally, being pulled upward toward the incision to facilitate the dissection and the placement of the endo-ATG45 stapler on the proximal bronchus. Izumi et al. [16] proved that granulation tissue formation at the bronchial stump is reduced after stapler closure comparison to suture closure. In order to investigate the role of PRP during the healing process of the bronchial stump, we decided to avoid suture closure. After the bronchial suture line had been dried with a sponge, it was covered with a cylindrical clot of PRP (Figure 1), applied vertically to the bronchial stump. All animals were carefully monitored during every postoperative day of observation. They were all sacrificed 4 weeks after the operation (Table 1).

**Figure 2:**
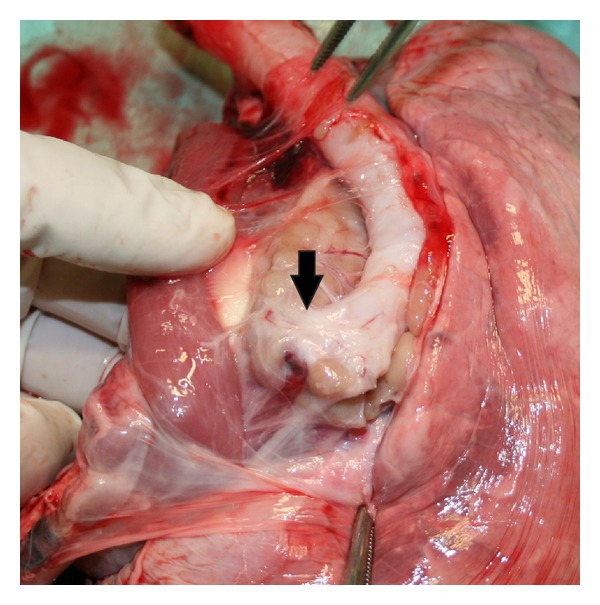
Animal necropsy. The trachea and the right lung are harvested. The arrow shows the bronchial stump suture line.

During necropsy, the bronchial stump, the operative field, and the entire thoracic cavities were thoroughly inspected (Figure 2). Then, the trachea with the right lung were harvested, routinely cut, and fixed in 10% buffered formalin solution.

### 2.5. Histological Examination and Immunohistochemical Analysis

The resected specimens were sent to the Laboratory of Pathology. Transverse sections of the bronchial stumps vertical to the line of the closure were made. Two sections per stump were taken. Before section preparation staples were carefully removed in all samples. The sections were embedded in paraffin, and four micron sections were made. These sections were stained with eosin and haematoxylin for morphological examinations. Using the optical microscope, the presence of granulation tissue was assessed in each sample and was quantified as the percentage of the total tissue area. The measurements from the two sections per animal were averaged. Histological assessment of the slides was performed by two independent, blinded to the treatment modalities, pathologists.

### 2.6. Statistical Analysis

Data are shown as percentages of the presence of granulation tissue per transverse section taken. The percentages in each group of animals were compared using Mann-Whitney *U* test. Statistical calculations were performed using Statistical package STATA 9.0 for Windows. All results with a two-sided *P* level ≤ 0.05 were considered statistically significant.

## 3. Results

**Figure 3:**
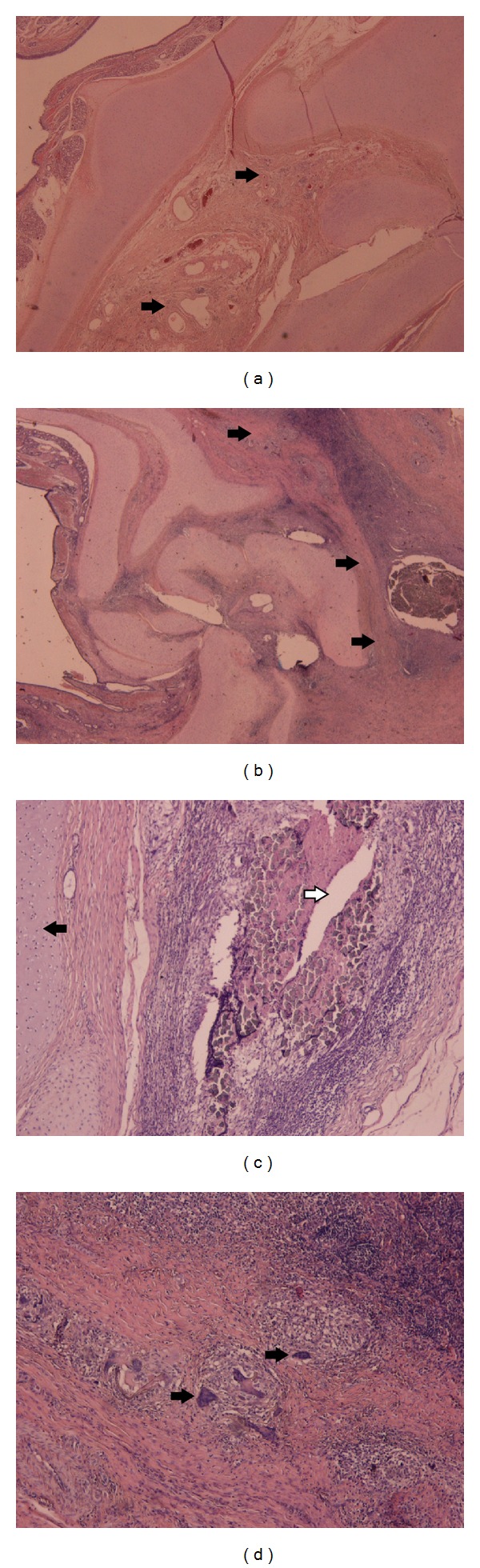
Eosin/Haematoxylin stain. Cross-section of the bronchial stump (×20) showing minimal granulation tissue formation (arrows) in the non-PRP group (a) and abundant granulation tissue formation in the PRP group (b). Black arrow defines cartilage, while white arrow illustrates a suture (c). Granulation tissue consisted of chronic inflammatory cells (d), including foreign body giant cells (arrows) forming granulomas (×200).

**Figure 4:**
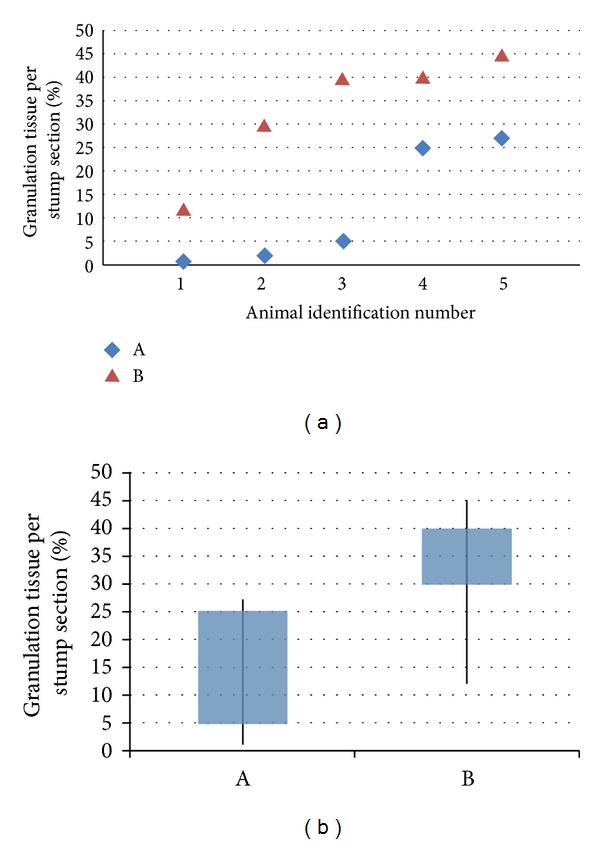
(a) Percentages of granulation tissue (%) per transverse section taken (from lowest to highest for each group). (b) The percentage is significantly higher in PRP group (group B). All results with a two-sided *P* level ≤ 0.05 were considered statistically significant.

Histologically, all samples displayed inflammatory reactions due to the bronchial closure technique. The granulation tissue in all cases consisted of chronic inflammatory cells, including foreign body giant cells forming granulomas (Figure 3). The bronchial cartilages at the stump were satisfactory aligned and the luminal side of the bronchial stump was lined by bronchial epithelium in all groups. The percentage of granulation tissue formed at the stumps was significantly higher in the PRP group of animals (Figure 4). This observation was confirmed when statistical analysis using Mann-Whitney *U* test was performed (*P* = 0.0268).

## 4. Discussion

Bronchial stump complications are commonly observed after pneumonectomy in humans. The prevention and treatment of these complications are important topics in thoracic surgery. A considerable number of retrospective studies have been performed on bronchial closure techniques in human medicine, and a few experimental studies have been carried out on the basis of these retrospective studies. However, bronchial stump closure techniques, their histological healing patterns, and possible bronchial stump complications after pneumonectomy need further experimental and clinical investigation.

A new strategy to promote the healing cascade is to prepare a substance for application to the bronchial stump. The ideal substance in pulmonary surgery is one that possesses several properties such as the ability to adhere to the tissue [17]. It should have a good biocompatibility, without provoking any tissular lesion or affecting the healing process. It should be biodegradable, with no risk of infectious transmission, and should not stimulate adhesion formation, which makes any reoperation difficult. Finally, it should be easy to use. Most of these adhesive substances are only mechanical occluders, and they do not have any positive biological effects. They are also applied usually through postoperative procedures like, for example, the bronchoscopic occlusion of a fistula with a collagen screw plug [18]. On the other hand, autologous PRP is a safe, feasible, and reliable new healing promoter with important therapeutic effects that is used intraoperatively [13–15]. It is easily produced with minimal basic equipment and may be useful in certain types of acute and chronic wounds, although the timing and optimum number of applications requires further study [12].

### 4.1. How PRP Works

PRP functions as a tissue sealant and drug delivery system, with the platelets initiating wound repair by releasing locally acting growth factors via *α*-granules degranulation [19]. The secretory proteins contained in the *α*-granules of platelets include platelet-derived growth factor (PDGF-AA, BB, and AB isomers) and many others [20–24], which aid healing by attracting undifferentiated cells in the newly formed matrix and triggering cell division. PRP may suppress cytokine release and limit inflammation, interacting with macrophages to improve tissue healing and regeneration, promote new capillary growth, and accelerate epithelialization in chronic wounds [25].

When platelets are activated, they release their growth factors almost immediately and continue to synthesize additional growth factors for several days, after which they lose vitality [26]. The cytokine response generated by PRP fulfills the requirements for appropriate healing: it promotes immediate onset but does not persist to cause chronic inflammation.

## 5. Conclusion

The main findings of this study are as follows: PRP application is a feasible and reliable technique not associated with any negative local or systemic effect. In thoracic surgery and especially lung resection operations, the application of PRP triggers the granulation process and promotes healing through an earlier initiation of the inflammatory response.

## Figures and Tables

**Table 1:** Parameters of the experiment.

*n*	G	GT%	S	PRP	AW (kgr)		OD (min)	IC	P		
					D1	DoE			BT	AD	C
1	A	1	M	−	25	31,4	90	−	Fever	++	−
2	A	2	M	−	26,2	32,7	93	−	<39°C	−	−
3	A	5	M	−	26	31,8	92	−	<39°C	+	−
4	A	25	M	−	26,8	30,3	88	−	<39°C	−	−
5	A	27	M	−	26,2	31,3	95	−	Fever	+	−

1	B	12	M	+	26,5	34,8	106	−	<39°C	−	−
2	B	30	M	+	25,7	33,5	102	−	<39°C	−	−
3	B	40	M	+	26	33,6	97	−	<39°C	−	−
4	B	40	M	+	25,5	33	98	−	<39°C	−	−
5	B	45	M	+	25,8	34,1	100	−	<39°C	−	−

*n*: postoperative identification number (from lowest to highest granulation tissue percentage); G: group; GT%: percentage of granulation tissue per stump section (%); S: sex; PRP: platelet-rich plasma; AW: animal weight; D1: day one; OD: operative duration; DoE: date of euthanasia; IC: intraoperative complications; P: postoperative; BT: body temperature; AD: animal discomfort; C: complications.

## References

[1] Graham EA, Singer JJ (1933). Successful removal of an entire lung for carcinoma of the bronchus. *Journal of the American Medical Association*.

[2] Wilkins EW, Scannell JG, Craver JG (1978). Four decades of experience with resections for bronchogenic carcinoma at the Massachusetts General Hospital. *Journal of Thoracic and Cardiovascular Surgery*.

[3] Al-Kattan K, Cattalani L, Goldstraw P (1994). Bronchopleural fistula after pneumonectomy with a hand suture technique. *Annals of Thoracic Surgery*.

[4] Pietrzak WS, Eppley BL (2005). Platelet rich plasma: biology and new technology. *Journal of Craniofacial Surgery*.

[5] Sommerer D, Süß R, Hammerschmidt S, Wirtz H, Arnold K, Schiller J (2004). Analysis of the phospholipid composition of bronchoalveolar lavage (BAL) fluid from man and minipig by MALDI-TOF mass spectrometry in combination with TLC. *Journal of Pharmaceutical and Biomedical Analysis*.

[6] Hotchkiss JR, Sanders MH, Clermont G, Crooke PS (2007). Preventing “bored-lung disease” when treating patients with ventilatory failure. *Critical Care Medicine*.

[7] Glenny RW, Bernard SL, Luchtel DL, Neradilek B, Polissar NL (2007). The spatial-temporal redistribution of pulmonary blood flow with postnatal growth. *Journal of Applied Physiology*.

[8] Doctor A, Price B, Bhargava N, Dicanzio J, Arnold JH (2001). High-frequency oscillatory ventilation of the perfluorocarbon-filled lung: dose-response relationships in an animal model of acute lung injury. *Critical Care Medicine*.

[9] Budas GR, Churchill EN, Mochly-Rosen D (2007). Cardioprotective mechanisms of PKC isozyme-selective activators and inhibitors in the treatment of ischemia-reperfusion injury. *Pharmacological Research*.

[10] Tomos P, Felekouras E, Poupalou A (2009). Video-assisted lung resection using radiofrequency ablation in a porcine model. *Journal of Surgical Research*.

[11] Nakakuki S (1994). Bronchial tree, lobular division and blood vessels of the pig lung. *The Journal of Veterinary Medical Science*.

[12] Gonshor A (2002). Technique for producing platelet-rich plasma and platelet concentrate: background and process. *International Journal of Periodontics and Restorative Dentistry*.

[13] Lacci KM, Dardik A (2010). Platelet-rich plasma: support for its use in wound healing. *Yale Journal of Biology and Medicine*.

[14] Mehta S, Watson JT (2008). Platelet rich concentrate: basic science and current clinical applications. *Journal of Orthopaedic Trauma*.

[15] Weibrich G, Kleis WKG, Kunz-Kostomanolakis M, Loos AH, Wagner W (2001). Correlation of platelet concentration in platelet-rich plasma to the extraction method, age, sex, and platelet count of the donor. *International Journal of Oral and Maxillofacial Implants*.

[16] Izumi Y, Kawamura M, Gika M, Nomori H (2010). Granulation tissue formation at the bronchial stump is reduced after stapler closure in comparison to suture closure in dogs. *Interactive Cardiovascular and Thoracic Surgery*.

[17] Feito BA, Rath AM, Longchampt E, Azorin J (2000). Experimental study on the in vivo behaviour of a new collagen glue in lung surgery. *European Journal of Cardio-Thoracic Surgery*.

[18] Tao H, Araki M, Sato T (2006). Bronchoscopic treatment of postpneumonectomy bronchopleural fistula with a collagen screw plug. *Journal of Thoracic and Cardiovascular Surgery*.

[19] Harrison P, Cramer EM (1993). Platelet *α*-granules. *Blood Reviews*.

[20] Eppley BL, Woodell JE, Higgins J (2004). Platelet quantification and growth factor analysis from platelet-rich plasma: implications for wound healing. *Plastic and Reconstructive Surgery*.

[21] Knighton DR, Ciresi KF, Fiegel VD (1986). Classification and treatment of chronic nonhealing wounds: successful treatment with autologous platelet-derived wound healing factors (PDWHF). *Annals of Surgery*.

[22] Knighton DR, Doucette M, Fiegel VD, Ciresi K, Butler E, Austin L (1988). The use of platelet derived wound healing formula in human clinical trials. *Progress in Clinical and Biological Research*.

[23] Pietramaggiori G, Kaipainen A, Czeczuga JM, Wagner CT, Orgill DP (2006). Freeze-dried platelet-rich plasma shows beneficial healing properties in chronic wounds. *Wound Repair and Regeneration*.

[24] Robson MC, Phillips LG, Thomason A (1992). Recombinant human platelet-derived growth factor-BB for the treatment of chronic pressure ulcers. *Annals of Plastic Surgery*.

[25] Marx RE (2004). Platelet-rich plasma: evidence to support its use. *Journal of Oral and Maxillofacial Surgery*.

[26] Heldin C-H, Westermark B (1999). Mechanism of action and in vivo role of platelet-derived growth factor. *Physiological Reviews*.

